# In Thyroidectomized Thyroid Cancer Patients, False-Positive I-131 Whole Body Scans Are Often Caused by Inflammation Rather Than Thyroid Cancer

**DOI:** 10.1177/2324709616633715

**Published:** 2016-02-25

**Authors:** Yana Basis Garger, Mathew Winfeld, Kent Friedman, Manfred Blum

**Affiliations:** 1 NYU Langone Medical Center, NY, USA

**Keywords:** thyroid cancer, papillary thyroid cancer, inflammation, whole body scan, WBS, 131-iodine, PTC, false positive

## Abstract

*Objective.* To show that I-131 false-positive results on whole-body scans (WBSs) after thyroidectomy for thyroid cancer may be a result of inflammation unassociated with the cancer. *Methods.* We performed a retrospective image analysis of our database of thyroid cancer patients who underwent WBS from January 2008 to January 2012 to identify and stratify false positives. *Results.* A total of 564 patients underwent WBS during the study period; 96 patients were referred for 99 I-131 single-photon emission computed tomography (SPECT/CT) scans to better interpret cryptic findings. Among them, 73 scans were shown to be falsely positive; 40/73 or 54.7% of false-positive findings were a result of inflammation. Of the findings, 17 were in the head, 1 in the neck, 4 in the chest, 3 in the abdomen, and 14 in the pelvis; 1 had a knee abscess. *Conclusions.* In our series, inflammation caused the majority of false-positive WBSs. I-131 SPECT/CT is powerful in the differentiation of inflammation from thyroid cancer. By excluding metastatic disease, one can properly prognosticate outcome and avoid unnecessary, potentially harmful treatment of patients with thyroid cancer.

## Introduction

In a thyroidectomized patient, scintillation scanning of the whole body (WBS) after radioactive iodine administration is the optimal way to identify the physical location of persistent or recurrent local and metastatic thyroid cancer. We have previously shown that I-131 single-photon emission computed tomography (SPECT/CT) may properly confirm the nature of cryptic WBS isotopic foci as thyroid cancer or demonstrate sites that are not thyroid cancer. Understanding this major difference can reduce unnecessary, improper treatment with I-131.^1^

We now demonstrate that many previously indeterminate areas of clinical concern are actually attributable to inflammation.

## Methods

This is a HIPAA-compliant, NYU Langone Medical Center Institutional Review Board–approved study that was designed to amplify our 2011 study.^1^ We performed a retrospective image analysis of our initial database of 182 patients who had 184 WBSs and added 382 more patients, for a total of 564 thyroid cancer patients who underwent WBS from January 2008 to January 2012. The examination was performed as part of standard endocrine management after a total thyroidectomy for differentiated thyroid cancer.

Thyroid-stimulating hormone (TSH) elevation was obtained either by withdrawing thyroid hormone or by administering recombinant TSH (rTSH, Thyrogen), per provider discretion. In all patients TSH was >40 mIU/mL. When T4 withdrawal was used, TSH and thyroglobulin were assayed before isotope administration, and in the rTSH group, the specimens were obtained on days 3 and 5 of the regimen, respectively. The patients were not pregnant or nursing. Our patient preparation, low iodine diet, bowel cleansing, imaging technique for planar WBS, and I-131 SPECT/CT image acquisition were described in detail in our 2011 publication.^1^ Ultrasonography of the neck or other diagnostic procedures were ordered, as clinically indicated, by the endocrinologist managing each patient. The results of these tests were correlated with our WBS SPECT/CT findings.

The planar WBS images were interpreted as part of clinical care. If the planar study was adequate for a confident interpretation of isotope localization, management of the patient proceeded without additional nuclear imaging. If, however, iodine localization was anatomically ambiguous or atypical on a planar WBS and, thus, defined as cryptic or indeterminate, an I-131 SPECT/CT was performed on the same or the next day. All the authors together reviewed all the planar and SPECT/CT images obtained in all patients who underwent SPECT/CT and recorded the location of cryptic foci related to inflammation. There was no disagreement among them.

We then compared the frequency of planar WBS studies referred for SPECT/CT in our patients who underwent WBS from August 2009 to January 2012 with the frequency of planar WBS studies referred for SPECT/CT in our January 2008 to August 2009 patient population.

## Results

**Figure 1.**
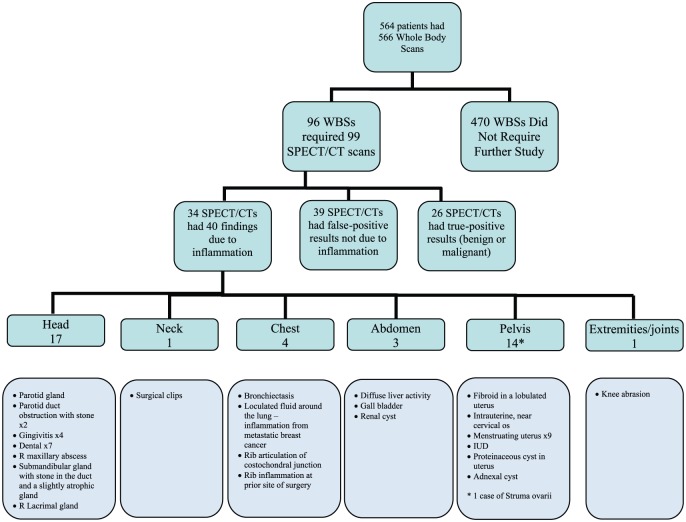
Schematic of patients who underwent WBSs and SPECT/CTs and the findings of those studies. Abbreviations: WBS, whole body scan; SPECT/CT, single-photon emission computed tomography; IUD, intrauterine device.

A schematic of the data is shown in Figure 1. A total of 566 WBSs were performed. Of these, 482 (85%) did not require further study because the identified I-131 foci were unanimously judged by the reading and reviewing nuclear and endocrine physicians to represent thyroid remnant tissue or thyroid cancer.

**Figure 2.**
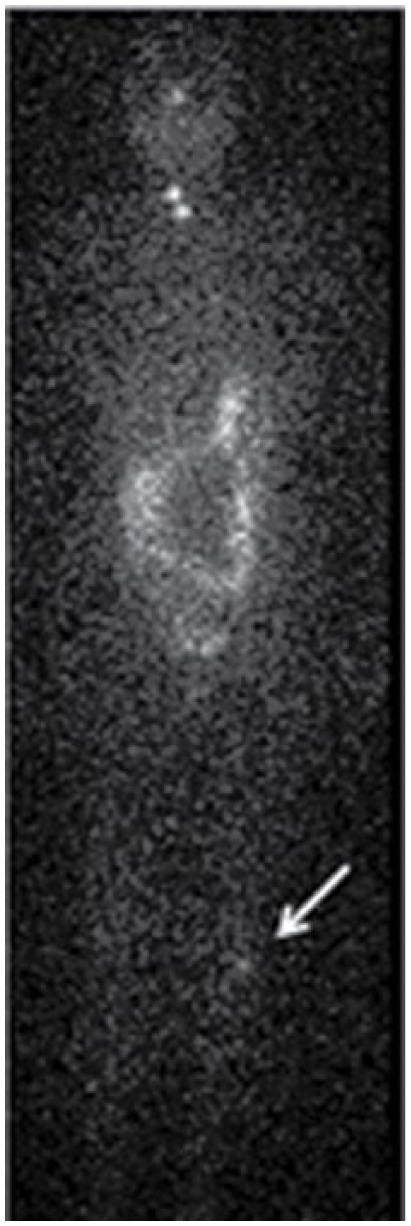
Asymmetrical left knee uptake on an 8-day posttherapy WBS: a 53-year-old woman with a history of papillary thyroid carcinoma (PTC) was treated with surgery and 100 mCi of I-131. WBS at 6 and 8 days posttherapy demonstrated persistent asymmetric uptake at the left knee. The patient had a clinical history of a knee abrasion several days previously. Physical exam demonstrated a small abscess at the site of the abrasion, which corresponded anatomically to the area of increased uptake on WBS. Abbreviations: WBS, whole body scan.

Generally, each patient with cryptic findings had only 1 cryptic focus and, therefore, a SPECT/CT of a single anatomical region was done. However, 3 patients had 2 obscure foci, and SPECT/CT of the 2 corresponding anatomical areas was performed. One additional patient underwent a posttherapy WBS that revealed increased, asymmetric uptake in the left knee (Figure 2). The patient had recently fallen, and physical examination revealed an abscess on the skin over the knee, which coincided with the area of uptake on WBS; SPECT/CT was not performed.

A total of 96 WBS patients were referred for 99 SPECT/CT scans because there were cryptic findings on the planar 131-I WBSs. Of the 96 WBSs, 73 were falsely positive, with 40/73 or 54.7% attributable to inflammation, based on supporting clinical data. In contrast, in 33/73 (45.2%), the SPECT/CT permitted the reading physicians to attribute the cryptic finding to physiological trapping of inorganic I-131 or otherwise metabolized iodine in nonthyroid organs.

**Figure 3.**
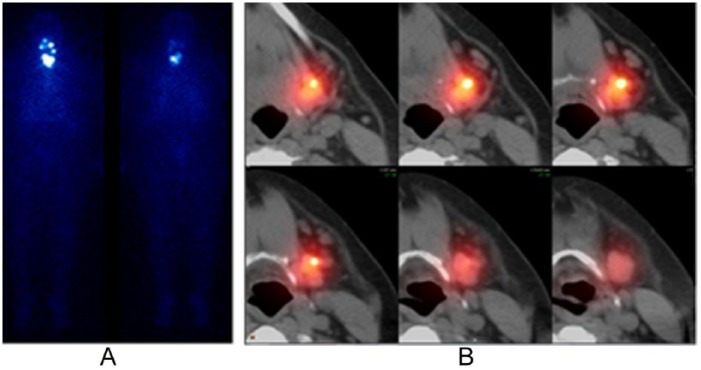
Parotid duct stone resulting in obstruction and inflammation as seen on WBS and SPECT/CT. A 50-year-old woman with a history of PTC was treated with surgery and 100 mCi of I-131. Posttherapy WBS (3A) demonstrates left-neck activity that on SPECT/CT (3B) corresponded to a stone impacted in the submandibular salivary gland duct. Abbreviations: WBS, whole body scan; SPECT/CT, single-photon emission computed tomography.

The anatomical sites of cryptic observations were distributed as follows. There were 17 patients who had increased uptake in the head, attributed to inflammation, based on SPECT/CT findings and then subsequently confirmed by clinical means. Some of these patients had more than 1 SPECT/CT cryptic focus in the head. Also, 3 patients had unilateral parotid gland swelling and 131-I uptake. In 2 of these patients, SPECT/CT demonstrated a stone in the parotid duct resulting in obstruction and inflammation (Figure 3). Another patient had right lacrimal gland inflammation; 11 patients had dental and/or gingival inflammation. One patient was found to have several spots of cryptic uptake in the neck without other evidence of thyroid cancer, such as thyroglobulin elevation. SPECT/CT showed evidence of inflammation surrounding surgical clips.

**Figure 4.**
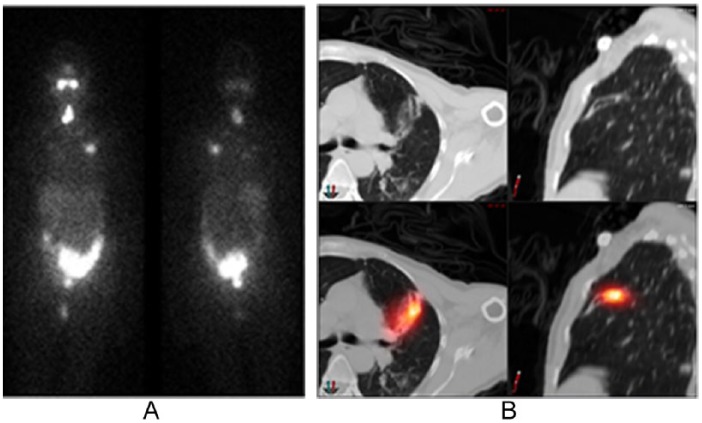
Cryptic uptake in the chest, corresponding to bronchiectasis on SPECT/CT. A 56-year-old woman with a history of PTC treated with surgery and then 100 mCi of I-131. Posttherapy WBS (4A) demonstrates focal activity in the lungs. On SPECT/CT (4B), this area was diagnosed as bronchiectasis. Abbreviations: WBS, whole body scan; SPECT/CT, single-photon emission computed tomography.

Four patients had focal, cryptic uptake in the chest that corresponded to inflammation. One patient was found on SPECT/CT to have bronchiectasis (Figure 4). The second patient had a history of breast cancer, and SPECT/CT showed loculated pleural fluid that was clinically determined to be inflammation resulting from metastatic breast cancer effusion. In 2 patients, the SPECT/CT localized the uptake to the ribs: one at the site of rib surgery and the other associated with costochondritis.

**Figure 5.**
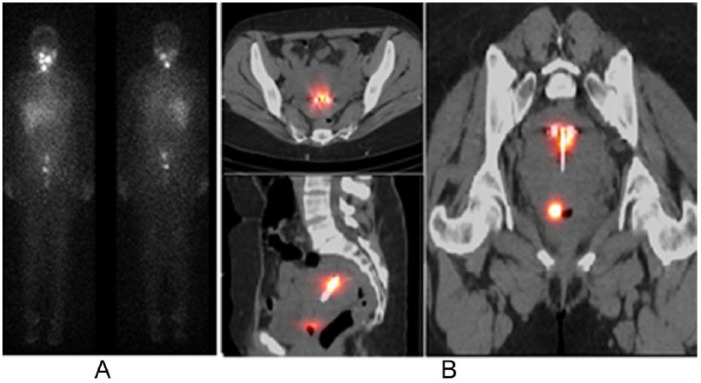
Inflammation around an intrauterine device and at a menstruating uterus/cervix as seen on WBS and SPECT/CT. A 41-year-old woman with a history of PTC was treated with surgery and then 150 mCi of I-131. Posttherapy WBS (5A) demonstrates 2 foci in the pelvis that on SPECT/CT (5B) were confirmed to represent accumulation of I-131 in the uterine cavity associated with an intrauterine device and at the cervix. Abbreviations: WBS, whole body scan; SPECT/CT, single-photon emission computed tomography.

In all, 14 patients had pelvic cryptic findings that SPECT/CT elucidated as inflammation. In 9, there was I-131 uptake within the uterine cavity at the time of menstruation. One each had increased uptake in a fibroid, around an intrauterine device (IUD; Figure 5), a uterine cyst, and an adnexal cyst. Additionally, there was 1 struma ovarii.

## Discussion

WBSs are commonly performed after thyroidectomy and then subsequently in the course of thyroid cancer management. WBSs are used to determine the extent of local residual/recurrent disease and metastases and for dosimetry for radioactive iodine therapy. Unfortunately, as with all studies, WBSs may be subject to errors of interpretation, and there is a tendency to accept any region that accumulates I-131 as apparent thyroid cancer, especially if it is located outside of the neck. Consequently, misinterpretation of a WBS may lead to improper staging, unnecessary administration of radioactive iodine, improper management, emotional stress, and superfluous financial burden.

We have shown that nonthyroid cancer I-131 uptake in WBS may coincide with inflammation. In this communication, we speak about inflammation in its widest sense that encompasses a spectrum of closely regulated mechanisms. It may be a response to local irritation, infection, tissue injury, or ill-understood forces that involve global bodily systems.

Hyperemia, vasodilation, local edema, and increased capillary permeability permit tissue access not only to cells and proteins that are normally confined to blood vessels, but can also cause WBS isotope I-131 accumulation. Taken together, all the causes of inflammation combine to be one of the most prevalent causes of false-positive results on WBS.

Glazer et al^2^ in 2013, as well as Shapiro et al^3^ in 2000, summarized the literature of false-positive results on WBS. Many of the false positives were not located in regions that are known to harbor organified iodine. Rather, they often correspond to inorganic iodine localizing sites.^4-6^ Although inflammation is listed as one of many such nonthyroidal causes of false positives on WBS, it was not considered prevalent. In contrast, we have observed inflammation as the cause of 54.7% of false positives in all areas of the body, which is a major and most important difference between the current and prior investigations and publications.

The female pelvis is often the site of inflammation.^7-9^ The onset of a menstrual cycle results in a cascade of inflammatory mediators that lead to tissue destruction and menstruation, but also inflammation.^10^ In uterine fibroid tissue, proinflammatory factors are significantly increased.^11-13^ IUDs are known to induce an intense inflammatory response, and this is the primary mechanism of their effectiveness as contraceptive agents.^14-16^ Several pelvic pathological lesions, which can present in our patient population, including microglandular uterine hyperplasia and endometriosis, have been found to have histological findings of chronic inflammation.^17^ Human ovulation and rupture of a follicle from the surface of the ovary leads to a local inflammatory cascade) in the ovary.^18-20^ Ovarian pathology in which inflammatory processes have been described include ovarian cancer and autoimmune oophoritis, which is associated with polycystic ovaries.^18,21-23^ Each of these processes can contribute to false-positive findings on WBS.

False-positive I-131 activity is also seen in the skeleton and extremities. Knees frequently appear symmetrically bright on WBS. In 1 case (Figure 2), asymmetry was related to recent physical trauma and subsequent infection. Inflammation in other joints—temporomandibular joint dysfunction syndrome (TMJ), the spine, ribs, and the pelvis—can appear as false positives.

Perioral inflammation secondary to gingivitis, dental abscesses, and sialoadenitis is a relatively common occurrence, which on WBS can be misinterpreted as pathological uptake in the head or cervical lymphatic metastases. The parotid and other salivary glands take up iodine and quickly clear it. However, when there is outflow obstruction from a stone or inflammation, iodine will accumulate, but its excretion may be retarded. Our salivary gland findings are most likely a result of a combination of salivary iodine trapping and inflammatory sialoadenitis attributable to 131-I therapy radiation injury.

One patient in our series was initially difficult to classify. She had received I-131 and was found to have focal activity on the right side of the oral cavity. SPECT/CT at that time localized the lesion to the maxilla, but we could not convincingly determine whether metastatic disease or dental inflammation was the cause. One year subsequently, the patient was found to have a dental abscess in the area of the previously noted focus of RAI uptake. It is noteworthy that at that time, dental curetting did not reveal thyroid cells on histological examination. Initially, she may have had inflammation (infection) that was not yet clinically apparent but became more prominent over time. Alternatively, she may have had a malignant focus in the maxilla, which after administration of therapeutic radioiodine became inflamed and underwent necrosis. Although it is impossible to know the sequence of the events that occurred, it is important to consider that an inexplicable finding on a WBS may be an as-yet clinically undiscovered inflammatory process.

The postsurgical neck, with postoperative inflammation, is a source of false-positive findings on WBS. Unfortunately, SPECT/CT can be misleading because the metal of the surgical clips or suture material may appear bright on CT. Clinical history can help guide the differential diagnosis.

Bronchiectasis (Figure 4), pulmonary infections, primary neoplasms, metastases, or effusions may cause false-positive WBS in the chest. Therefore, to avoid a false diagnosis and inappropriate therapy with I-131, we recommend SPECT/CT when a nonthyroid neoplasm or pneumonic infection are clinical considerations.

Many abdominal areas are locations for false-positive I-131 activity. We found increased I-131 WBS uptake at the site of a renal cyst in a patient who had no other evidence of metastatic thyroid cancer, and the level of thyroglobulin was not elevated. Renal cysts are thought to be bright on WBS because of renal clearance of iodine, which subsequently is sequestered in the cyst. However, recent evidence suggests that inflammation plays a key role in the development of renal cysts and, thus, may lead to additional false positives on WBS.^24^ Hepatic cysts, cholecystitis, or diverticulosis may also cause false positives.

We commonly perform WBS on women of child-bearing age while they are menstruating in an effort to be most certain they are not in early pregnancy, a time when chorionic gonadotrophins may not be sufficiently elevated to achieve a positive result on screening tests. Whereas preferentially performing WBS at the time of menstruation may be considered an iatrogenic source of false-positive results, it clearly demonstrates that hyperemia secondary to this physiological cause commonly leads to increased pelvic uptake on WBS.

It is noteworthy that in our prior analysis of 184 WBSs from January 2008 to August 2009, 21.7% had cryptic findings and went on to a SPECT/CT examination. In contrast, in the subsequent 397 WBSs from August 2009 to January 2012, findings were cryptic in only 14.6% (n = 58) of the scans. This reduction in cryptic findings may be attributable to having learned from our prior experience. We learned that inflammation was a common source of false-positive I-131 uptake on WBS. Thus, we reduced the number of SPECT/CT scans that were needed for proper diagnosis. Nevertheless, spurious results caused by inflammation were still a significant contributor to false-positive findings, accounting for 54.7% of false-positive WBSs in our study of 566 WBS.

The major limitation of our study is that it was conducted at a single institution. Additionally, the lower rate of SPECT/CT in the latter part of our trial somewhat obscures the frequency of false positivity resulting from inflammation. Although it can be considered a limitation that there was no preplanned protocol for whom to refer for further SPECT/CT imaging, we believe that this is a strength of our study because it mirrors the reality of clinical practice.

The CT portion of a SPECT/CT of the abdomen/pelvis results in a total effective radiation dose of 4.9 mSv, which is roughly double the amount of yearly exposure patients receive from background radiation. Although this increase in exposure to irradiation is not prohibitively high, it is not insignificant. It is, however, less in terms of both radiation and cost than with other imaging modalities, such as PET/CT. The risks are counterbalanced by the benefit that the SPECT/CT can help elucidate cryptic findings and, thus, avoid unnecessary, much greater exposure from radioiodine therapy.

## Conclusion

In conclusion, the WBS not only shows areas of iodide uptake resulting from thyroid cells, but also reveals iodide accumulation that is mediated by the mechanisms of inflammation. The 2 processes should not be confused. As we have shown, inflammatory processes are quite prevalent in patients undergoing WBS.
